# Impact of Motivational Enhanced Adherence Counseling and Point-of-Care Viral Load Monitoring on Viral Load Outcome in Women on Life-Long ART: A Randomized Pilot Study

**DOI:** 10.1155/2022/4887202

**Published:** 2022-09-05

**Authors:** Mercy T. Mutambanengwe-Jacob, Charles C. Maponga, K. Rivet Amico, Bernard Ngara, Nonhlanhla Yende-Zuma, Tariro D. Chawana, Teacler Nematadzira, Justice F. Gumbo, Tendayi Goverayi, Petronella Matibe, Bernadette V. Malunda, Jim Aizire, Taha E. Taha, Mary G. Fowler, Lynda Stranix-Chibanda

**Affiliations:** ^1^Department of Pharmacy and Pharmaceutical Sciences, Faculty of Medicine and Health Sciences, University of Zimbabwe, Harare, Zimbabwe; ^2^University of Zimbabwe Clinical Trials Research Centre, Harare, Zimbabwe; ^3^Department of Health Behavior and Health Education, School of Public Health, University of Michigan, Ann Arbor, MI, USA; ^4^Department of Primary Health Care Sciences, Faculty of Medicine and Health Sciences, University of Zimbabwe, Harare, Zimbabwe; ^5^Centre for the AIDS Program of Research in South Africa (CAPRISA), Durban, South Africa; ^6^Department of Epidemiology, The Johns Hopkins Bloomberg School of Public Health, Baltimore, MD, USA; ^7^Department of Pathology, The Johns Hopkins Medicine, Baltimore, MD, USA; ^8^Child and Adolescent Health Unit, Faculty of Medicine and Health Sciences, University of Zimbabwe, Harare, Zimbabwe

## Abstract

We piloted the combined effectiveness of point-of-care viral load monitoring plus motivational enhanced adherence counseling (intervention) compared with routine care (control) in women identified at risk of virologic failure in the PROMOTE study in Zimbabwe. In an unblinded randomized study, consenting women with last viral load ≥200 copies/ml and/or pill count outside 90–110% range were randomized 1 : 1 to receive the intervention or continue routine care, comprising laboratory-based VL monitoring and standard EAC, from trained nurses and counsellors. Viral load was measured 0, 3, 6, and 12 months after enrolment. We compared viral suppression <200 copies/ml at 6 and 12 months between the arms through Fisher's exact test and sought associated factors by logistic regression with a 95% confidence interval (CI). Between December 2018 and July 2019, 50 women were enrolled (25 intervention and 25 controls) and followed until November 2020. At entry, 60% of the women were virally suppressed, 52% intervention *vs*. 68% control arm. Viral suppression was balanced between the two arms (*p* value = 0.248). At month 6 post study entry (primary endpont), 64% of the women retained in care were virally suppressed, 54% intervention vs. 76% control arm (*p* value = 0.124). At month12 post study entry (secondary endpoint), 69% of the women retained in care were virally suppressed, 67% intervention vs. 71% control arm women (*p* value = 0.739). More intervention women completed all scheduled sessions by month 6. Control group women were more likely to be virally suppressed at both timepoints. Only 25% had treatment switch by 12 months. Despite intense adherence support and viral load monitoring, sustained viral suppression remained elusive in women identified at risk of viral failure. These findings highlight the continued need for effective adherence intervention for women with unsuppressed HIV viral loads, efficient treatment switch strategies, as well as drug level monitoring.

## 1. Introduction

Increasing access to antiretroviral therapy (ART) has led to significant success in the fight against the human immunodeficiency virus (HIV) pandemic, yet low adherence to life-long ART continues to undermine its effectiveness in preventing vertical HIV transmission. Previous research in sub-Saharan Africa suggests that ART adherence is particularly challenging during the peripartum period [1–3]. Enhanced adherence counseling (EAC) has been noted to have a significant role in viral resuppression among adults on ART, with viral suppression being achieved in 28–61% of clients post-EAC [4, 5]. However, interventions applied to date have had short-lived success. EAC sessions that have appropriate information result in patient motivation and equip women with the necessary behavioral skills to adhere to their treatment and often result in better health outcomes [6, 7]. A systematic review of the motivational Information-Motivation-Behavioral Skills model showed its potential strength in impacting adherence behavior [8].

HIV viral load (VL) testing became the gold standard for HIV treatment monitoring and confirmation of treatment failure in 2013 [9]. In addition to the early identification of treatment failure, the knowledge of VL is thought to motivate patients to adhere to treatment [4, 10]. HIV VL testing remains limited by logistic, infrastructural, and personnel-related deficiencies in resource-limited settings (RLS). Centralized laboratory-based HIV VL testing that is prevalent in RLS is compounded by patient challenges to return to the clinic and long result turnaround time (TAT) that can stretch for months [9, 11, 12]. It often results in delayed adherence intervention where required. Use of point-of-care (POC) testing presents a major benefit with rapid result delivery, expedited intervention where necessary, and improved viral suppression and retention in care, thus removing some barriers to achieving viral suppression [13].

In 2014, Zimbabwe adopted the 2013 World Health Organization (WHO) ART guidelines, which included life-long treatment for pregnant women with HIV (Option B+), with an aim to achieve the 90-90-90 UNAIDS goals by 2018 [14]. These local guidelines defined virologic failure as having a viral load >1000 copies/ml based on two consecutive viral load measurements three months apart with adherence support, following the first viral load test [15]. While the use of this cut-off limit has been associated with low risk of disease burden and a decrease in HIV transmission [16], vertical HIV transmission, drug resistance, and virologic failure remain more common in patients with persistent low-level viremia (50–1000 copies/ml) than those with sustained viral suppression, defined as <50 copies/ml [17, 18]. Given the set targets, Zimbabwe fell short of the 90-90-90 goals with an estimated 82% of people with HIV having suppressed viral loads in 2020 [19] and 64.5% in women aged 15–49 years [20].

To improve adherence monitoring and treatment outcomes in women on life-long ART, this study sought to assess the effect of POC HIV VL monitoring and motivational EAC (mEAC) compared with routine care on virological suppression rates in women with elevated HIV VL and at risk of elevated VL in the PROMOTE study in Zimbabwe. We aimed to assess whether adherence counseling sessions that use facets of the information, motivation, and behavior (IMB) model combined with real-time viral load feedback through POC testing are more effective in providing adherence skills required to achieve viral suppression at 6 months and maintain long-term adherence to ART measured at 12 months after intervention.

## 2. Materials and Methods

### 2.1. Trial Design

It was a randomized (1 : 1), unblinded, implementation science pilot study nested within the PROMOTE observational cohort being followed at three clinics in Zimbabwe [21].

### 2.2. Study Participants

Women targeted for enrolment in this study were study participants at PROMOTE sites in Zimbabwe, living with HIV and experiencing elevated VL (≥200 copies/ml) at a previous PROMOTE study visit or considered at risk of virologic failure. At-risk criteria were defined as VL ≥ 50 copies/ml for three consecutive visits at least 6 months apart or abnormal pill count (no pill count data, pill count below 90% or above 110% at the participant's last visit). Women were included if they were willing and able to provide informed consent to enrol in this substudy, had initiated an ART regimen at least six months prior to enrolment, were enrolled at a participating research site, and met the elevated VL or at-risk criteria. Women were excluded if they had plans to move out of the research area within one year.

### 2.3. Setting

The study took place at the three PROMOTE study sites in Zimbabwe, two located in Chitungwiza, a dormitory city located about 30 km away from Zimbabwe's capital city of Harare, with a total of 354 women in the PROMOTE study, and one in Harare with 94 women. Participants included in this study were a subset of former participants from the multicountry IMPAACT PROMISE randomized trial (IMPAACT 1077BF) (NCT01061151) [22, 23] and subsequently being followed in the PEPFAR-funded PROMOTE observational study in Zimbabwe [21] (Supplementary 1). Apart from Zimbabwe, the PROMOTE study was conducted at other participating sites in Malawi, South Africa, and Uganda.

ART initiated by these ART-naïve women during the PROMISE study for the purpose of preventing vertical HIV transmission during the pregnancy and breastfeeding period when they did not meet the criteria for initiating ART at that time is described elsewhere [22, 23]. These women were transitioned to life-long ART from 2015 in line with the release of the START study results [24]. During the PROMOTE study, women collected ART from their primary care provider and reported routinely for observational study visits every six months, where laboratory-based HIV VL test was conducted at each visit. If a woman fell pregnant, visits were conducted every eight weeks until delivery, then at week 6 postdelivery, month 6 postdelivery, and every six months thereafter. Women with viral loads ≥1000 copies/ml were referred to the Zimbabwe National ART Program for further management.

In the national program, people living with HIV who were on ART had routine VL testing at 6 months after ART initiation, and annually thereafter if considered stable, i.e., VL < 1000 copies/ml and no opportunistic infections. VL testing was done on dried blood spots or plasma samples at centralized referral laboratories. Patients with elevated VL > 1000 copies/ml were switched to second line treatment following a second VL > 1000 copies/ml with EAC sessions having been conducted, or where there was evidence of immunologic or clinical failure without a repeat VL result. Pregnant and breastfeeding women on ART had their first ANC VL either 3 months after ART initiation if ART naïve or at the first antenatal clinic (ANC) visit if already on ART. If the woman had an elevated VL > 1000 copies/ml, EAC was conducted with a repeat VL after one month. Pregnant and breastfeeding women were prepared for treatment switch if repeat VL remained elevated above 1000 copies/ml. Women in the PROMOTE study had access to these services while being followed up on the study.

### 2.4. Interventions

#### 2.4.1. Recruitment Strategy

Research records from 448 women enrolled in the PROMOTE study in Zimbabwe were reviewed. Participants meeting the eligibility criteria were selected for the substudy entry and approached by study personnel at a scheduled PROMOTE study visit.

#### 2.4.2. Study Procedures

After providing informed consent to enrol into the substudy, eligible women were enrolled and randomized 1 : 1 to receive either motivational EAC (mEAC) and POC VL testing (intervention arm) or standard of care (SOC) VL monitoring and EAC (control arm). Following randomization, blood samples for VL monitoring were collected by trained nurses at study entry and at months 3, 6, and 12 after enrolment. As no testing machines were housed at the clinics where the study was conducted, samples for both arms were sent to the University of Zimbabwe Clinical Trials Research Centre (UZ-CTRC) Central Laboratory, where testing was done using the Gene Xpert® for POC tests and the Abbott® for SOC tests by trained laboratory scientists. The Gene Xpert Qual Assay used for POC testing was validated against the qualitative COBAS® AmpliPrep/COBAS® TaqMan® HIV-1 Test version 2.0 at BARC Laboratory in South Africa prior to study implementation [25]. VL results were sent back to research sites via phone if same day result delivery was required, and/or followed by e-mail copies to site laboratories for result giving at contact after printing and filing in participant files. EAC or mEAC was conducted at the time of receipt of the participant's VL result and monthly for two months by trained counsellors for women with viral load ≥200 copies/ml in each arm using either a mEAC tool (intervention arm) or a standard guided tool (control arm) until the next viral load test. Participants were recommended for treatment switch if the viral load remained ≥200 copies/ml following two cycles of counseling and viral load testing at month 6. At month 9, viral load was done for women with persistent VL ≥ 200 copies/ml and at month 6, visit to assess VL was declined following treatment switch. All participants were followed up for 12 months.

Women who fell pregnant during the study could continue study participation as the benefits derived from the interventions included reduced transmission to the infants and better health outcomes for the mother and the baby.

### 2.5. Outcomes

The primary outcome of interest was the proportion of women with viral suppression at 6 months and sustained at 12 months poststudy entry. Viral suppression was defined as VL < 200 copies/ml at each of the two timepoints. The characteristics of women who continued to have VL ≥ 200 copies/ml (unsuppressed VL) were also determined at both time points. For our secondary outcomes, we determined if pill count eligibility criteria use was a good predictor of virologic failure, and whether the use of POC VL testing improved HIV VL result delivery to participants.

### 2.6. Sample Size Determination

Based on Pocock's formula, 25 participants were selected for each study arm, with an additional 3 (∼10%) to allow for loss to follow-up or reasons prohibiting intervention, providing 80% power to detect a 28% increase in the proportion of women who achieve the primary outcome of viral suppression at 6 months.

### 2.7. Randomization

A randomization list was generated by a trained data manager at one of the three study sites using Microsoft Excel. Continuous numbers between 0 and 1 were generated using the = RAND () function on a list of randomization arms, noted as Arm 1 (intervention) or Arm 2 (control). A list of study participant identification numbers linking the treatment assignments was generated for the study and the randomization list applied to the arms. Participants were enrolled at their respective sites and assigned the next available arm on the list consecutively by the data manager responsible for generating the list, with data teams from each site requesting a treatment assignment at the point of participant enrolment.

### 2.8. Data Management

Sociodemographic-, medical-, laboratory-, and drug-related data routinely collected for the PROMOTE study was extracted from the PROMOTE dataset. Additionally, a structured data form was designed to capture the study-specific viral load data and results of the implementation process. These forms were completed by trained study staff in real time, reviewed for completeness and accuracy, and captured into the online study database. Participants were considered lost to follow-up if they missed two or more visits, including the month 12 visit, or a visit was noted as missed if one was missed, including the month 12 visit.

### 2.9. Statistical Methods

#### 2.9.1. Descriptive Statistics

Quantitative data was analyzed using STATA, Version 15.1, Stata Corp, 4905 Lakeway Drive, College Station, Texas 77845, USA, [26] to estimate the proportion of women with viral suppression at month 6 and month 12. Categorical variables were summarized by frequency and proportions, while continuous variables were summarized as mean and standard deviation (SD) for normally distributed data or median and interquartile range (IQR) for skewed data. Continuous VL data was categorized into suppressed and unsuppressed using a viral load cut-off limit of 200 copies/ml.

#### 2.9.2. Predictive Model

For categorical data, Fisher's exact and McNemar tests were used to test for association between variables and randomization or viral suppression using the 95% confidence interval (CI) with a difference being noted if *p* value <0.05 for independent and dependent variables, respectively. For continuous data, a regression analysis was done to estimate the association between variables and randomization or viral suppression. The characteristics of the women who continued to have a viral load ≥200 copies/ml despite the intervention provided were analyzed using bivariate and multivariate logistic regression to determine the odds ratio (OR) and adjusted odds ratio (aOR) with 95% CI, respectively, at months 6 and 12. Variables used for aOR determination were selected based on prior literature [27]. Pill count as a predictor of viral suppression was determined using logistic regression, while frequencies of result turnaround times were noted for each arm.

### 2.10. Ethical Approval

The study was conducted in compliance with local regulatory requirements and in accordance with Good Clinical Practice (GCP) principles laid out by the International Conference on Harmonization (ICH) and the Declaration of Helsinki. The study was approved by the Medical Research Council of Zimbabwe (MRCZ) (Approval: MRCZ/B/1545) and the Joint Research Ethics Committee (JREC) for the University of Zimbabwe Faculty of Medicine and Health Sciences, and Parirenyatwa Group of Hospitals (Approval: JREC/201/18). Written informed consent was obtained from the women for their participation prior to the conduct of the study.

## 3. Results and Discussion

### 3.1. Results

#### 3.1.1. Screening and Enrolment

**Figure 1:**
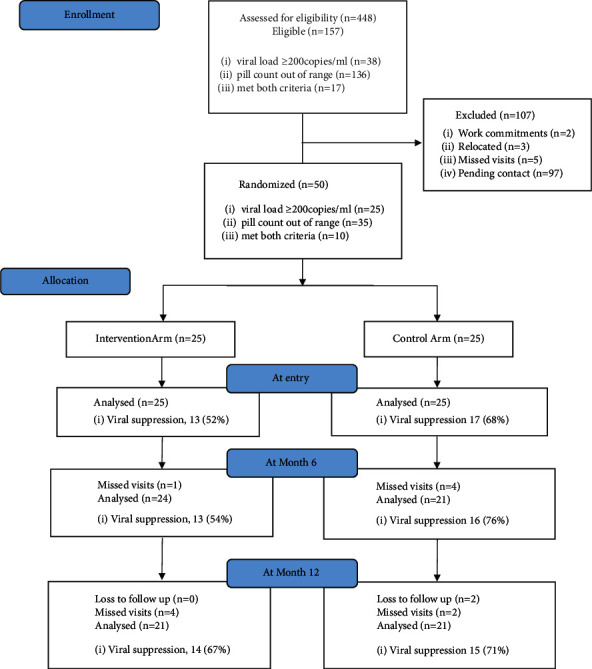
Summary of participant flow and viral suppression data.

Between December 2018 and July 2019, 448 women were screened from the PROMOTE study in Zimbabwe. 157 (35%) participants were eligible for entry into the substudy, with 410 (91.5%) being virally suppressed (Figure 1). From the eligible participants, 50 (31.8%) were enrolled into the substudy using purposive sampling, 25 in each arm. 107 participants were excluded from study enrolment for reasons including work commitments, relocation, missed prior visits, or study was fully accrued prior to contact. Of the 38 participants with a viral load ≥200 copies/ml at screening, 25 (65.8%) were enrolled, 14 in the intervention arm and 11 in the control arm. Of the 13 not enrolled, 1 (2.6%) cited work commitments, 2 (5.2%) had relocated, 4 (10.5%) had missed their PROMOTE study visits, and 6 (15.8%) were pending contact. 35 women were enrolled based on at-risk criteria by pill count, and of these, 10 met both high VL and at-risk criteria. Enrolled women were followed up until November 2020.

### 3.2. Baseline Characteristics of Enrolled Participants

Table 1summarizes the participants' baseline characteristics. Of the enrolled participants, mean age (SD) was 33.4 (5.7) years, mean (SD) duration on antiretroviral treatment was 3.4 (0.7) years, and a majority, 96%, were on first-line therapy with Tenofovir Disoproxil Fumarate/Lamivudine (TDF/3TC) backbone. For the 34 participants with pill count data, the mean (SD) pill count was 96.8% (14.3%). Of the enrolled participants, 30 (60%) were married, and 5 (10%) had a primary regular partner. Of these, 31 (88.6%) had disclosed their HIV status to the partner. Two participants (4%) were pregnant at study entry, while 10 (20%) were breastfeeding. Forty-one (82%) had up to secondary education, and only 7 (14%) were formally employed. Twenty-three (46%) took less than 30 minutes to get to the clinic, and depression score ranged from 0 to 20 with 45 (90%) participants recording a depression score below 5. Study arms were well-matched based on Fisher's exact test and regression analysis (*p* value >0.05).

### 3.3. Viral Load Outcome

At study entry, 30 (60%) of the women were virally suppressed, 13 (52%) in the intervention and 17 (68%) in the control arm (*p* value 0.387) (Figure 1). At month 6, 45 (90%) participants were retained in care, 29 (64%) were virally suppressed, 13 of 23 (54%) in the intervention arm and 16 of 21 (76%) in the control arm (*p* value 0.212). Five participants missed their month 6 visit. At month 12, 42 (84%) of the participants were retained in care. 29 (69%) were virally suppressed, 14 of 21 (67%) in the intervention arm and 15 of 21 (71%) in the control arm (*p* value 1.000). Two participants were considered lost to follow-up, while 6 missed the month 12 visit.

### 3.4. Characteristics of Women with Unsuppressed Viral Load

We considered the site of participation, randomization, age, duration of treatment, marital status, travel time to clinic, depression score above 4, mean pill count at screening, HIV disclosure, pregnancy intention at last pregnancy, breastfeeding status, level of education, and employment status at enrolment (Supplementary 2-3). At month 6, the odds of having viral suppression decreased by a factor of 0.37 in the intervention arm compared with women in the control arm (CI 0.10–1.34), while viral suppression decreased by a factor of 0.90 and 0.70 for every 1-year increase in age and duration on treatment, respectively. There were 5% lower odds of viral suppression with HIV status disclosure (Table 2).

There was a marginal change in the odds of viral suppression for participants in the intervention arm compared to those in the control arm after adjusting for age, duration on treatment, and disclosure status to the partner using multiple regression (aOR 0.38, CI 0.06–2.15) (Table 2).

At month 12, the odds of viral suppression decreased by a factor of 0.80 in the intervention arm compared with participants in the control arm (CI 0.22–2.97). Viral suppression decreased by a factor of 0.92 and 0.55 with each 1-year increase in age and duration on treatment, respectively. There was a 1.2-fold increase in viral suppression with HIV status disclosure, while suppression decreased by a factor of 0.35 with treatment switch (Table 3). After adjusting for age, duration on treatment, HIV disclosure status, treatment switch and randomization, and disclosure status, there was a 1.10-fold increase in viral suppression in participants in the intervention compared with women in the control arm (Table 3).

### 3.5. Pill Count and Viral Suppression

There was an association between participants eligible by pill count and viral load suppression at 6 months and 12 months poststudy entry. Participants who were eligible for enrolment based on pill count were likely to be virally suppressed by 98% at month 6 (OR 0.02, CI 0.001–0.16) (Supplementary 2), and 86% at month 12 (OR 0.14, CI 0.03–0.63) (Supplementary 3). Average pill count at entry was not associated with unsuppressed VL, OR 1.03 (CI 0.97–1.08) at month 6 (Supplementary 2) and sustained unsuppressed VL at month 12 OR 1.01 (CI 0.96–1.07) (Supplementary 3).

### 3.6. Result Turnaround Time

Table 4 gives result delivery times and shows whether the result delivery was on time for each arm. The VL result was considered on time when given on day 0 for participants in the intervention (POC) arm and at the next contact for participants in the control (laboratory-based) arm. Result delivery was on time for all participants in the control arm, save for the month 3 visit in a participant with a missed visit. The main reason for results delivery failure on Day 0 for the intervention arm was delays from the laboratory 13/13 (100%) at enrolment, 7/11 (64%) at month 3, 15/20 (75%) at month 6, and 12/17 (71%) at month 12 visit. Other reasons noted included participants not willing to wait or missed tests.

### 3.7. EAC Sessions Conducted and Full Intervention Delivery

#### 3.7.1. Full Intervention Delivery

**Figure 2:**
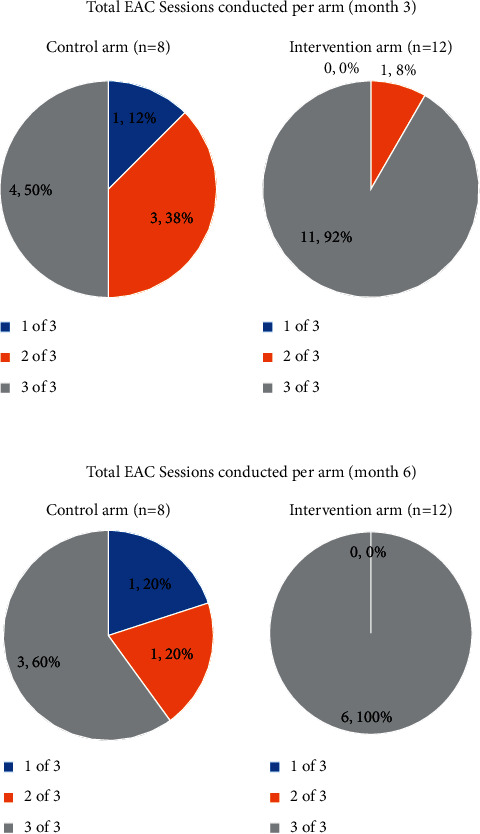
Number of EAC sessions conducted for participants with VL ≥ 200 copies/ml according to arm.

Participants with VL ≥ 200 copies/ml at entry or month 3 were required to attend three EAC or mEAC sessions before the next VL blood sample was taken at month 3 or 6, respectively. Of the 20 participants with VL ≥ 200 copies/ml at enrolment, 15 (75%) had all three EAC sessions with a follow-up VL test (full intervention) at month 3 visit. In the control arm, 4 (50%) of the 8 participants received the full intervention by month 3 visit, and in the intervention arm, 11 (92%) of the 12 participants received the full intervention. At month 6, nine of the 11 participants (91%) not suppressed (VL ≥ 200 copies/ml) at month 3 received the full intervention by the month 6 visit. In the control arm, 3 (60%) of the 5 participants not suppressed at month 3 visit received the full intervention at month 6, while in the intervention arm, all 6 (100%) of the participants with unsuppressed VL at month 3 received the full intervention (Figure 2).

#### 3.7.2. Total EACs Sessions

Of the 192 EAC visits required from study entry up to the month 12 visit, 155 (81%) of the participants with VL ≥ 200 copies/ml managed to complete their required EAC sessions. Of the 155, 56 VL visits were conducted, and 10 results showed viral suppression, 5 at month 3, 2 at month 6, and 3 at month 12 visit (Table 5).

#### 3.7.3. Total VL Visits

Participants were required to attend three VL testing visits at months 3, 6, and 12 poststudy entry. From a total of 150 required VL visits, 126 (84%) viral load visits were conducted, with 86 (68%) of the 126 VL results obtained showing viral suppression. Ten participants had a sustained VL ≥ 200 copies/ml from study enrolment through to the month 12 visit (Table 6).

#### 3.7.4. Visit Burden and Viral Suppression

Visit burden at month 6 was assessed for both women with suppressed and unsuppressed VL (Table 7). Participants with VL ≥ 200 copies/ml had a median of 5 EAC/mEAC and VL visits compared with 4 visits for those with VL < 200 copies/ml, and 3 visits for those who missed the month 6 visit.

### 3.8. Treatment Switch

Fourteen (28%) participants had their treatment switched by the month 12 visit, 7 (50%) in each arm. Ten were switched as part of the change in national treatment guidelines from Efavirenz (EFV)-based ART to Dolutegravir (DTG)-based ART, while four were switched because of treatment failure. Of the 10 having regimen switch because of a change in national guidelines, one had elevated VL at month 6 and became virally suppressed by month 12 visit. Of the four participants switched because of treatment failure, one participant was switched at the month 2 visit and became virally suppressed by month 3 visit before missing the month 6 visit and ultimately being lost to follow up by the month 12 visit. The new regimen was not documented.

Of the 16 women with VL ≥ 200 copies/ml at month 6, three (19%) had the ARV treatment switch to second line treatment because of virologic failure. The timing of treatment switch varied with one participant being switched at the month 6 visit, the second between 91 and 180 days, and the third was switched more than 180 days after the month 6 visit. All three participants were switched from TDF/3TC/EFV to second line treatment consisted of either Abacavir (ABC) (two participants) or Zidovudine (AZT) (one participant) plus Lamivudine (3TC) backbone, with ritonavir boosted Atazanavir (ATVr). Of these three, only two presented for the month 12 visit.

## 4. Discussion

In this pilot study aimed at achieving viral suppression using a VL cut-off limit of <200 copies/ml, IMB-modeled motivational EAC sessions with POC VL testing did not reduce the proportion of women with unsuppressed VL at 6 months and 12 months poststudy entry. Apart from being eligible based on pill count, there were no characteristics that were significantly associated with suppressed VL at both 6 months and 12 months poststudy entry. Pill count did not predict virologic failure, as participants who were eligible based on pill count were more likely to be suppressed at both month 6 and 12 poststudy entry. POC VL result delivery achieved better outcome in terms of full EAC delivery. Result delivery for the intervention arm was not on time for more than half the participants.

Different studies have reported various suppression rates with different VL cut-off limits being used, indicating the need to review the VL limit used in care as low-level viremia continues to be associated with transmission at different levels [9, 16, 28]. This study used a viral load cut-off limit of <200 copies/ml. Although set lower than the WHO VL cut-off limit, the viral suppression rate noted among women on ART who were either failing ART or classified as at risk of failing treatment in this study fell below the target 90% requirement to meet the UNAIDS 90-90-90 goals [29]. Viral suppression is required to ensure the prevention of HIV transmission and the development of drug resistance among patients on ART. The intervention increased VL suppression from 52% at entry to 54% at month 6 and 67% at month 12. While it was not significant compared with the control, a larger increase from baseline viral suppression was noted in the intervention arm compared with the control arm by the month 12 visit, indicating promise toward sustained viral suppression with the shorter mEAC tool, if implemented in a larger sample. Limited effectiveness in increasing viral suppression levels to above the target of 90% for both intervention and control arms was likely attributed to some similarities in the intervention and SOC tools used for EAC. Local guidelines changed prior to study implementation, and both methods were motivational in nature [15, 30]. Upon comparing suppression rates in this study with baseline suppression among the same cohort of women being followed up in the PROMOTE study across all sites in sub-Sahara Africa using the same cut-off limit of 200 copies/ml, viral suppression was higher at 86% in the larger group and 90.8% among participants from Zimbabwe, compared with 91.5% at screening this subset of women [21], showing the evidence of effectiveness of good intervention strategies within this cohort being actively followed up over a long period in a well-resourced clinical trial setting. Among other studies looking at adherence among women, a study conducted in Uganda and South Africa looking at adherence to ART among pregnant and postpartum women during the Option B+ era showed viral suppression (VL < 400 copies/ml) above 86% among these peripartum women in Uganda but lower rates of 57% in South Africa [1]. Local survey results conducted in Zimbabwe showed HIV viral suppression (VL < 1000 copies/ml) of 76.8% among women aged 15–49 years, indicating lower viral suppression among women in the country and in the region compared to women in general [31]. These results could not be compared because of the different cut-off limits used.

We looked at treatment switch following unsuppressed viral load up to 6 months poststudy entry, as part of a strategy to achieve viral suppression following virologic failure. This study noted a treatment switch rate of 25% among those participants needing a regime change, raising the need to explore further why participants were not accessing treatment switch when required. In 2019, the WHO updated its guidelines to shift from EFV-based ART to DTG, combined with two nucleoside reverse-transcriptase inhibitors (NRTIs) as first-line treatment because of the evidence of higher viral suppression and lower risk of discontinuing treatment and developing HIV drug resistance compared with EFV-based regimens among treatment-naive adults [32]. Other advantages of using DTG over EFV include lower potential for drug–drug interactions, more rapid viral suppression, and a higher genetic barrier to developing HIV drug resistance [32]. Zimbabwe revised its 2016 guidelines to incorporate this regimen in its ART policy, which reflected in this study as 10 of the 50 participants were switched to DTG-based ART during the study even though they were virally suppressed. One participant who had an elevated VL was also switched to DTG and subsequently suppressed, indicating the benefit of early treatment switch to a more potent regimen in women with persistently elevated VL.

Various factors have been associated with unsuppressed viral load in different settings [1, 2, 7, 17, 27, 33]. The characteristics assessed for association with viral suppression used in this study were based on the baseline data collected in the PROMOTE study. Of these, the ones selected for the adjusted odds ratio based on those characteristics found to be significantly associated with viral suppression in literature are as follows: age [1, 3, 34], duration on treatment [2, 27], and disclosure to partner or partner support [1, 2, 7, 35]. Randomization, pill count eligibility, as well as treatment switch were additional characteristics explored in this study as they were required to address the study objectives. Among the characteristics explored, pill count was the only factor found to be protective of viral suppression.

Pill count was used as one of the eligibility criteria to assess if a pill count out of the range of 90–110% was associated with future virologic failure. The results indicate that the pill count was close to 100% for those assessed, and those who were eligible based on pill count were more likely to remain suppressed. Atuhaire et al. assessed self-report and not pill count as a possible proxy for virologic failure [27] and noted its role in adherence assessment. This study indicates that pill count may not be a reliable proxy, contrary to what was observed by Achieng et al. [36]. It is likely because of different practices in collecting pill count data across the three recruiting sites. While Seke North and St. Mary's CRSs routinely conducted pill counts for their participants, Harare Family Care CRS did not require their participants to bring their pills at each visit, and hence, the pill count was not conducted routinely.

The WHO have dropped the threshold for action from 1000 copies/ml with EAC now due with detectable VL (≥50 copies/ml), whilst also advocating for POC VL testing to reduce result TAT and provide real-time feedback to women on ART [9]. In this study, POC VL monitoring offered an opportunity for increased access to care for women failing treatment. As evidenced by studies done in Zimbabwe and within the region, POC VL monitoring is feasible and increases access to VL and retention in care and opportunity to improve VL outcome [10, 13, 37–39]. In some studies, POC VL and near-POC VL monitoring was also associated with good treatment switch ranging from 46%–86% [39, 40].

Result TAT was an important factor in monitoring viral response because of the early detection and reporting of virologic failure and action on results. In the intervention arm, result delivery on the day of testing (Day 0) was very challenging with less than half of the participants receiving the result on the same day of test. It was because of the POC testing machine being located off site and at the central laboratory. The study had access to one testing machine only, which was shared across the three research sites and among other studies. It resulted in near-POC services being provided with results being sent to sites via telephone or e-mail once ready or printed copies being sent the day following the visit to ensure that the participant received the result as soon as possible. Because of the effort to get results to sites as soon as possible, participants in the POC arm received their results for a median of 25.5 days of testing, compared with 130 days median in the control arm. There is, therefore, value in utilizing regionalized testing machines as the result TAT was still reduced with participants having earlier access to results with the less complex 2-hour VL testing platform.

There was a trend observed toward higher completion of the mEAC cycle in the intervention arm compared to the control arm participants, indicating that the three elements of the intervention are highly relevant to the current efforts to adapt WHO 2021 treatment guidelines locally and across the region [9]. A study by Bvochora among the general population receiving ART in Harare showed no association between the number of EAC sessions attended and viral suppression among people with a repeat VL test [41]. While adherence support is essential, consideration should be given to the maximum benefits of EAC versus visit burden among this cohort, as women are often involved in other activities, given the 60% noted employment rate for formal and self-employment combined. Visit burden was considered in terms of total number of visits attended by each participant. Participants with unsuppressed VL were likely to have more visits. Hence, a higher burden of visits was noticed compared to those with a suppressed VL or those who missed the month 6 visit. Further exploration is required using qualitative data to determine whether monthly EAC visits pose a concern in terms of visit burden on providers and intervention recipients.

This study's findings begin to outline implementation considerations for treatment experienced perinatal women that need additional focus and modification of current practice. Policy should consider using POC testing to ensure rapid result delivery and intervention in women with unsuppressed HIV VL.

As gaps remain in the effort to achieve viral suppression through adequate counseling and treatment switch strategies, there is a need to explore increased access to drug level monitoring and resistance testing to ascertain sooner the reasons for poor viral suppression in women who have been on ART for a prolonged period. Reasons for failure to switch treatment timeously also need exploring as there is an increased risk of development and transmission of drug resistance virus. There are still other factors that impact adherence to ART that were not explored in this study, including the impact of individual compared to group adherence counseling, person delivering the intervention, and children under 5 years being cared for by the woman. These factors have been associated with unsuppressed VL in some studies and could be considered a contributor in this cohort of women.

### 4.1. Limitations

Because of the small sample size used in this study, we were not able to detect significant differences in interventions and comprehensively conclude on the characteristics predictive of future virologic failure in women on life-long ART. The results are also limited in being generalizable as the cohort of women selected for this study were in follow-up in an ongoing research study with compensation for attending visits for at least three years. The study was also not able to measure fidelity in intervention delivery as each participating site delivered both interventions to their participants. There is a risk of intervention contamination as a counsellor could easily administer an incorrect intervention or viral load test being requested on the wrong platform resulting in delays in the POC arm. The interventions in both arms were also delivered as a comprehensive package. Hence, we were not able to detect the effect of each component of the intervention on viral suppression.

## 5. Conclusion

Adherence support and rapid intervention in unsuppressed viral load are vital in women on life-long ART as viral suppression remained low in this cohort of treatment experienced women. The use of POC is recommended to improve result delivery. More research is required to explore strategies that will improve adherence to ART in this cohort. The use of more impactful motivational EAC with limited contact time could be explored. The availability of qualitative data can also help tease out relevant intervention strategies that may be more effectively implemented.

## Figures and Tables

**Table 1:** Summary of baseline characteristics.

Variable	Intervention (*n* = 25)	Control (*n* = 25)	Total (*n* = 50)	*p* value
Age, mean (SD)	33.3 (5.7)	33.6 (5.9)	33.4 (5.7)	0.846

Breast feeding status				0.725
Yes	6 (24)	4 (16)	10 (20)	
No	19 (76)	21 (84)	40 (80)	

Depression score, mode (range)	0 (0–5)	0 (0–20)	0 (0–20)	0.733

Depression score, *n* (%)				0.349
0 to 4	21 (84)	24 (96)	45 (90)	
5 and above	4 (16)	1 (4)	5 (10)	

Duration on treatment-years, mean (SD)	3.3 (0.7)	3.4 (0.7)	3.4 (0.7)	0.579

Education level reached				1.000
Primary	4 (16)	5 (20)	9 (18)	
Secondary	21 (84)	20 (80)	41 (82)	

Employment status, *n* (%)				1.000
Formally employed	4 (16)	3 (12)	7 (14)	
Not employed	10 (40)	10 (40)	20 (40)	
Self-employed	11 (44)	12 (48)	23 (46)	

HIV disclosure, *n* (%)				0.190
Yes	14 (56)	17 (68)	31 (62)	
No	4 (16)	0 (0)	4 (8)	
No data	7 (28)	8 (32)	15 (30)	

Marital status, *n* (%)				1.000
Married	15 (60)	15 (60)	30 (60)	
Not married	10 (40)	10 (40)	20 (40)	

Pill count, mean (SD)	94 (15)	99 (13)	96.8 (14.3)	0.281

Pregnant, *n* (%)				1.000
Yes	1 (4)	1 (4)	2 (4)	
No	24 (96)	24 (96)	48 (96)	

Pregnancy intention, *n* (%)				1.000
Not intended	12 (48)	12 (48)	24 (48)	
Not sure	2 (8)	1 (4)	3 (6)	
Yes, intended	11 (44)	12 (48)	23 (46)	

Site, *n* (%)				0.238
St. Mary's CRS	15 (60)	9 (36)	24 (48)	
Seke North CRS	6 (24)	11 (44)	17 (34)	
Harare Family Care CRS	4 (16)	5 (20)	9 (18)	

Therapy, *n* (%)				0.490
TDF/3TC/EFV	23 (92)	24 (96)	47 (94)	
TDF/3TC/LPVr	2 (8)	0 (0)	1 (2)	
AZT/3TC/NVP	0 (0)	1 (4)	2 (4)	

Time to clinic, *n* (%)				0.317
<30 min	9 (36)	14 (56)	23 (46)	
30-60 min	10 (40)	8 (32)	18 (36)	
>1 hour	6 (24)	3 (12)	9 (18)	

Viral load at screening, *n* (%)				0.572
<200 copies/ml	11 (44)	14 (56)	25 (50)	
≥200 copies/ml	14 (56)	11 (44)	25 (50)	

**Table 2:** Factors associated with viral suppression at 6 months.

Variable (*n* = 45)	Odds ratio (95% CI)	*p* value	Adjusted odds ratio (95% CI)	*p* value
Arm, *n* (%)				
Intervention (mSOC)	Ref		Ref	
Control (SOC)	0.37 (0.10–1.34)	0.129	0.38 (0.07–2.15)	0.273

Age (years), mean (SD)	0.90 (0.79–1.00)	0.056	0.86 (0.72–1.04)	0.126

Duration on treatment (years), mean (SD)	0.70 (0.30–1.65)	0.416	1.32 (0.37–4.67)	0.664

HIV disclosure, *n* (%)				
Not disclosed	Ref		Ref	
Disclosed	0.95 (0.08–11.9)	0.967	1.37 (0.07–25.38)	0.832

**Table 3:** Factors associated with viral suppression at 12 months.

Variable (*n* = 42)	Odds ratio (95% CI)	*p* value	Adjusted OR (95% CI)	*p* value
Arm, *n* (%)				
Intervention (mSOC)	Ref			
Control (SOC)	0.80 (0.22–2.97)	0.739	1.10 (0.19–6.44)	0.916

Age (years), mean (SD)	0.92 (0.82–1.04)	0.182	0.95 (0.81–1.12)	0.570

Duration on treatment (years), mean (SD)	0.55 (0.22–1.35)	0.191	0.61 (0.17–2.19)	0.451

HIV disclosure, *n* (%)				
Not disclosed	Ref		Ref	
Disclosed	1.2 (0.11–13.32)	0.882	1.26 (0.08–19.09)	0.869

Treatment switch, *n* (%)				
No	Ref		Ref	
Yes	0.35 (0.06–1.87)	0.218	0.23 (0.02–2.34)	0.215

**Table 4:** Result delivery time per arm.

Intervention arm (POC testing)			Control arm (laboratory testing)		
Full	Result on time (day 0) *n*, (%)	Median, days (IQR)	Full	Result on time (at next contact) *n*, (%)	Median, days (IQR)
Enrolment (*N* = 25)	12 (48)	0 (0–1)	Enrolment (*N* = 25)	25 (100)	31 (29–35)
Month 3 (*N* = 18)	7 (39)	1 (0–11)	Month 3 (*N* = 21)	20 (95)	28.5 (27–82.5)
Month 6 (*N* = 23)	3 (13)	25.5 (2.5–105)	Month 6 (*N* = 21)	21 (100)	130 (58–179)
Month 12 (*N* = 21)	4 (19)	7 (2–21)	Month 12 (*N* = 20)	20 (100)	14 (8–127)

**Table 5:** EAC visit attendance for participants with VL ≥ 200 copies/ml at enrolment.

	Required visits			VL status for conducted visits	
	Visits conducted, *n* (%)	Missed visits, *n* (%)	Total visits required	VL < 200 copies/ml, *n* (%)	VL ≥ 200 copies/ml, *n* (%)
Month 0	20 (100)	0 (0)	20	0 (0)	20 (100)
Month 1	19 (95)	1 (5)	20		
Month 2	15 (75)	5 (25)	20		
Month 3	15 (75)	5 (25)	20	5 (33)	10 (67)
Month 4	11 (55)	5 (45)	16		
Month 5	10 (50)	6 (50)	16		
Month 6	18 (90)	2 (10)	20	2 (0)	16 (100)
Month 7	12 (75)	4 (25)	16		
Month 8	10 (63)	6 (37)	16		
Month 9	15 (94)	1 (6)	16		
Month 12	13 (88)	2 (12)	16	3 (23)	10 (77)
Overall	155 (81)	37 (19)	192	10 (15)	56 (85)

**Table 6:** Viral suppression based on VL visit attendance.

	Required visits				VL status for conducted visits	
	Baseline VL	Visits conducted, *n* (%)	Missed visits, *n* (%)	Total	VL < 200 copies/ml, *n* (%)	VL ≥ 200 copies/ml, *n* (%)
Month 3	VL ≥ 200 copies/ml	15 (75)	5 (25)	20	5 (67)	10 (33)
	VL < 200 copies/ml	24 (80)	6 (20)	30	23 (96)	1 (4)
	Total	39 (78)	11 (22)	50	28 (72)	11 (28)

Month 6	VL ≥ 200 copies/ml	10 (91)	1 (9)	11	0 (0)	10 (100)
	VL < 200 copies/ml	26 (93)	2 (7)	28	24 (92)	2 (8)
	Missed visits	9 (82)	2 (18)	11	5 (56)	4 (44)
	Total	45 (90)	5 (10)	50	29 (64)	16 (36)

Month 12	VL ≥ 200 copies/ml	13 (81)	3 (19)	16	3 (23)	10 (77)
	VL < 200 copies/ml	26 (90)	3 (10)	29	24 (92)	2 (8)
	Missed visits	3 (60)	2 (40)	5	2 (67)	1 (33)
	Total	42 (84)	8 (16)	50	29 (69)	13 (31)

Overall		126 (84)	24 (16)	150	86 (68)	40 (32)

**Table 7:** Visit burden at month 6 visit.

	Median, visits	(IQR)
VL ≥ 200 copies/ml (*n* = 16)	5	(3–6)
VL < 200 copies/ml (*n* = 29)	4	(3–5)
Missed month 6 visit (*n* = 5)	3	(1–5)
Total (*n* = 50)	4	(3–5)

## Data Availability

Data utilized and assessed in the present study are available from the corresponding author on request.

## Supplementary Materials

Supplementary Materials Supplementary 1: PROMOTE study design. Supplementary 2: table of factors associated with viral suppression at month 6. Supplementary 3: table of factors associated with viral suppression at month 12.
