# Supplementary material for: Impact of Motivational Enhanced Adherence Counseling and Point-of-Care Viral Load Monitoring on Viral Load Outcome in Women on Life-Long ART: A Randomized Pilot Study
Source: AIDS Res Treat. 2022 Sep 5;2022:4887202. doi: 10.1155/2022/4887202 (PMC9467808; doi:10.1155/2022/4887202)
Supplement: Supplementary Materials — Supplementary 1: PROMOTE study design. Supplementary 2: table of factors associated with viral suppression at month 6. Supplementary 3: table of factors associated with viral suppression at month 12. [file 4887202.f1.zip › Supplementary 1 - PROMOTE Study Design.pdf]

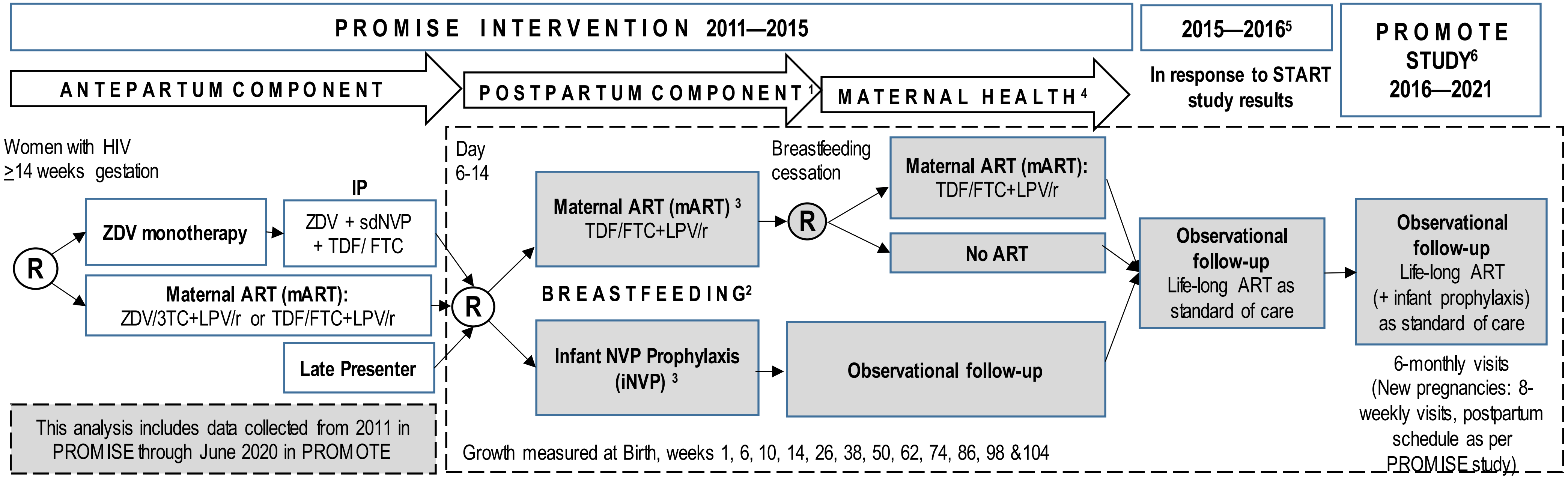

1. PROMISE inclusion criteria—*Maternal*: intent to breast feed, CD4 count ≥350 cells/mm^3 or country specific threshold for initiation of treatment if this threshold is >350 (within 30 days prior to entry), acceptable laboratory values hemoglobin, WBC, ANC, Platelets, ALT and estimated creatinine clearance. *Infant*: alive and healthy and not HIV infected, <14 days of age, acceptable laboratory values (within 14 days of birth) for hemoglobin, WBC, ANC, Platelets and ALT. Exclusion criteria—*Maternal*: current or history of TB (excluding positive PPD test or requirement for antiretroviral therapy for own health. *Infants*: birth weight <2kg. Infants received standard-of-care Cotrimoxazole prophylaxis >6 weeks and were followed for 104 weeks.
2. Randomized Postpartum ARV regimen continued through breastfeeding cessation or 18 months of age, whichever came first, or according to country guidelines. Breastfeeding duration determined by the mother.
3. Infants received standard-of-care nevirapine prophylaxis from birth for 6 weeks, and cotrimoxazole prophylaxis according to country guidelines.
4. Only mothers assigned to mART who did not meet local guidelines for ART initiation at cessation of breastfeeding underwent further randomization in PROMISE.
5. PROMISE intervention phase held June 2015 following release of START* study results and all women offered life-long ART as standard-of-care and remained in observational follow-up until the last mother-infant pair completed week 104 visit. Women transitioned to non-study ART prior to trial exit June 2016.
6. PROMOTE inclusion criteria—*Maternal*: Prior PROMISE study participation at a site selected for PROMOTE. *Infant/Child*: Prior PROMISE study participation at a site selected for PROMOTE or born to a mother enrolled in PROMOTE after study entry.

* INSIGHT START Study Group, *NEJM* 2015 http://dx.doi.org/10.1056/NEJMoa1506816

Abbreviations: ANC—absolute neutrophil count; ALT—alanine transaminase; AP—Antepartum; ART—antiretroviral treatment; ARVs—antiretroviral drugs; FTC—emtricitabine; g—grams; IP—Intrapartum; LPV/r—ritonavir boosted lopinavir; mg—milligrams; MH—Maternal Health; NVP—nevirapine; PP—Postpartum; R—randomization; sdNVP—single dose nevirapine; TB—tuberculosis; TDF—tenofovir diphosphate; WBC—white blood cell; ZDV—Zidovudine; 3TC—lamivudine
