# Supplementary material for: Impact of Motivational Enhanced Adherence Counseling and Point-of-Care Viral Load Monitoring on Viral Load Outcome in Women on Life-Long ART: A Randomized Pilot Study
Source: AIDS Res Treat. 2022 Sep 5;2022:4887202. doi: 10.1155/2022/4887202 (PMC9467808; doi:10.1155/2022/4887202)
Supplement: Supplementary Materials — Supplementary 1: PROMOTE study design. Supplementary 2: table of factors associated with viral suppression at month 6. Supplementary 3: table of factors associated with viral suppression at month 12. [file 4887202.f1.zip › Supplementary 2 - Table of factors associated with viral suppression at month 6 (1).docx]

Supplementary 2: Table of factors associated with viral suppression at month 6

| **Variable**  **(n=45)** | **VL < 200 copies/ml n=29** | **VL ≥ 200 copies/ml n=16** | **Odds Ratio**  **(95% CI)** | **p-value** |
| --- | --- | --- | --- | --- |
| Age (years), mean (SD) | 35.1 (5.7) | 31.5 (5.6) | 0.90 (0.79-1.00) | 0.056 |
| Arm, n (%) |  |  |  |  |
| Intervention (mSOC) | 13 (45) | 11 (69) | Ref |  |
| Control (SOC) | 16 (55) | 15 (31) | 0.37 (0.10-1.34) | 0.129 |
| Breastfeeding status, n (%) |  |  |  |  |
| No | 23 (79) | 13 (81) | Ref |  |
| Yes | 6 (21) | 3 (19) | 0.88 (0.19-4.14) | 0.876 |
| Depression score, n (%) |  |  |  |  |
| 0 to 4 | 28 (97) | 14 (88) | Ref |  |
| 5 and above | 1 (3) | 2 (13) | 4.00 (0.33-48.00) | 0.274 |
| Duration on treatment (years), mean (SD) | 3.4 (0.7) | 3.2 (0.8) | 0.70 (0.30-1.65) | 0.416 |
| Education level attained, n (%) |  |  |  |  |
| Primary level | 6 (21) | 1 (6) | Ref |  |
| Secondary level | 23 (79) | 15 (94) | 3.91 (0.43-35.83) | 0.227 |
| Employment status, n (%) |  |  |  |  |
| Formally employed | 4 (14) | 3 (19) | Ref |  |
| Not employed | 10 (34) | 7 (44) | 0.93 (0.16-5.54) | 0.939 |
| Self-employed | 15 (52) | 6 (38) | 0.53 (0.09-3.13) | 0.437 |
| HIV disclosure status, n (%) |  |  |  |  |
| Not disclosed | 2 (7) | 1 (6) | Ref |  |
| Disclosed | 19 (66) | 9 (56) | 0.95 (0.08-11.9) | 0.967 |
| No regular partner | 8 (28) | 6 (38) | - |  |
| Last pregnancy intention, n (%) |  |  |  |  |
| Not intentional | 14 (48) | 8 (50) | Ref |  |
| Not sure if it was intended | 1 (3) | 1 (6) | 1.75 (0.10-32.0) | 0.706 |
| Intentional | 14 (48) | 7 (44) | 0.88 (0.25-3.07) | 0.835 |
| Marital status, n (%) |  |  |  |  |
| Not married | 9 (31) | 8 (50) | Ref |  |
| Married | 20 (69) | 8 (50) | 0.45 (0.12-1.58) | 0.213 |
| Pill count (%), mean (SD) | 95 (17.8) | 100 (4.5) | 1.03 (0.97-1.08) | 0.364 |
| Pill count eligibility at screening, n (%) |  |  |  |  |
| Not eligible | 1 (3) | 11 (69) | Ref |  |
| Eligible | 28 (97) | 5 (31) | 0.02 (0.001-0.16) | <0.001 |
| Site, n (%) |  |  |  |  |
| St Mary’s CRS | 13 (45) | 9 (56) | Ref |  |
| Seke North CRS | 14 (48) | 3 (19) | 0.31 (0.07-1.40) | 0.128 |
| Harare Family Care CRS | 2 (7) | 4 (25) | 2.89 (0.43-19.28) | 0.273 |
| Time to clinic, n (%) |  |  |  |  |
| <30 min | 14 (48) | 8 (50) | Ref |  |
| 30-60 min | 10 (34) | 4 (25) | 0.70 (0.16-2.98) | 0.629 |
| >1 hour | 5 (17) | 4 (25) | 1.40 (0.28-6.77) | 0.676 |
