# Supplementary material for: Impact of Motivational Enhanced Adherence Counseling and Point-of-Care Viral Load Monitoring on Viral Load Outcome in Women on Life-Long ART: A Randomized Pilot Study
Source: AIDS Res Treat. 2022 Sep 5;2022:4887202. doi: 10.1155/2022/4887202 (PMC9467808; doi:10.1155/2022/4887202)
Supplement: Supplementary Materials — Supplementary 1: PROMOTE study design. Supplementary 2: table of factors associated with viral suppression at month 6. Supplementary 3: table of factors associated with viral suppression at month 12. [file 4887202.f1.zip › Supplementary 3 - Table of factors associated with viral suppression at month 12 (1).docx]

Supplementary 3: Table of factors associated with viral suppression at month 12

| **Variable**  **(n=42)** | **VL <200 copies/ml n=29** | **VL >200 copies/ml n=13** | **Odds Ratio**  **(95% CI)** | **p-value** |
| --- | --- | --- | --- | --- |
| Age (years), mean (SD) | 34.5 (5.7) | 31.8 (6.1) | 0.92 (0.82-1.04) | 0.182 |
| Arm, n (%) |  |  |  |  |
| Intervention (mSOC) | 14 (48) | 7 (54) | Ref |  |
| Control (SOC) | 15 (52) | 6 (46) | 0.80 (0.22-2.97) | 0.739 |
| Breastfeeding status, n (%) |  |  |  |  |
| No | 25 (86) | 8 (62) | Ref |  |
| Yes | 4 (14) | 5 (38) | 3.91 (0.84-18.17) | 0.082 |
| Depression score, n (%) |  |  |  |  |
| 0 to 4 | 27 (93) | 11 (85) | Ref |  |
| 5 and above | 2 (7) | 2 (15) | 2.45 (0.30-19.68) | 0.398 |
| Duration on treatment (years), mean (SD) | 3.4 (0.7) | 3.1 (0.9) | 0.55 (0.22-1.35) | 0.191 |
| Education level attained, n (%) |  |  |  |  |
| Primary level | 6 (21) | 1 (8) | Ref |  |
| Secondary level | 23 (79) | 12 (92) | 3.13 (0.34-29.09) | 0.316 |
| Employment status, n (%) |  |  |  |  |
| Formally employed | 3 (10) | 3 (23) | Ref |  |
| Not employed | 12 (41) | 5 (38) | 0.42 (0.06-2.81) | 0.369 |
| Self-employed | 14 (48) | 5 (38) | 0.35 (0.05-2.38) | 0.288 |
| HIV disclosure status, n (%) |  |  |  |  |
| Not disclosed | 3 (10) | 1 (7) | Ref |  |
| Disclosed | 20 (69) | 8 (62) | 1.2 (0.11-13.32) | 0.882 |
| No regular partner | 6 (21) | 4 (31) | - |  |
| Last pregnancy intention, n (%) |  |  |  |  |
| Not intended | 15 (52) | 7 (54) | Ref |  |
| Not sure | 1 (3) | 0 (0) | - |  |
| Intended | 13 (45) | 6 (46) | 0.99 (0.26-3.70) | 0.987 |
| Marital status, n (%) |  |  |  |  |
| Not married | 8 (28) | 5 (38) | Ref |  |
| Married | 21 (72) | 8 (62) | 0.61 (0.15-2.43) | 0.483 |
| Pill count (%), mean (SD) | 96.4 (17) | 98.9 (8.3) | 1.01 (0.96-1.07) | 0.688 |
| Pill count eligibility at screening, n (%) |  |  |  |  |
| Not eligible | 4 (14) | 7 (54) | Ref |  |
| Eligible | 25 (86) | 6 (46) | 0.14 (0.03-0.63) | 0.01 |
| Site, n (%) |  |  |  |  |
| St Mary’s CRS | 11 (38) | 7 (54) | Ref |  |
| Seke North CRS | 14 (48) | 2 (15) | 0.22 (0.04-1.30) | 0.096 |
| Harare Family Care CRS | 4 (14) | 4 (31) | 1.57 (0.29-8.42) | 0.598 |
| Time to clinic, n (%) |  |  |  |  |
| <30 min | 13 (45) | 8 (62) | Ref |  |
| 30-60 min | 10 (34) | 3 (23) | 0.49 (0.10-2.32) | 0.367 |
| >1 hour | 6 (21) | 2 (15) | 0.54 (0.08-3.36) | 0.511 |
| Time to treatment switch (days), mean (SD) | 201.4 (90.6) | 176.5 (303.3) | 1.00 (0.99- 1.01) | 0.786 |
| Treatment switch, n (%) |  |  |  |  |
| No | 19 (66) | 11 (85) | Ref |  |
| Yes | 10 (34) | 2 (15) | 0.35 (0.06-1.87) | 0.218 |
